# Fibroblast growth factor rescues brain endothelial cells lacking presenilin 1 from apoptotic cell death following serum starvation

**DOI:** 10.1038/srep30267

**Published:** 2016-07-22

**Authors:** Miguel A. Gama Sosa, Rita De Gasperi, Patrick R. Hof, Gregory A. Elder

**Affiliations:** 1 General Medical Research Service, James J. Peters Department of Veterans Affairs Medical Center, Bronx, New York, USA; 2 Department of Psychiatry, Icahn School of Medicine at Mount Sinai, New York, New York, USA; 3 Friedman Brain Institute, Icahn School of Medicine at Mount Sinai, New York, New York, USA; 4 Research and Development Service, James J. Peters Department of Veterans Affairs Medical Center, Bronx, New York, USA; 5 Fishberg Department of Neuroscience, Icahn School of Medicine at Mount Sinai, New York, New York, USA; 6 Department of Geriatrics and Palliative Care, Icahn School of Medicine at Mount Sinai, New York, New York, USA; 7 Neurology Service, James J. Peters Department of Veterans Affairs Medical Center, Bronx, New York, USA; 8 Department of Neurology, Icahn School of Medicine at Mount Sinai, New York, New York, USA

## Abstract

Presenilin 1 (Psen1) is important for vascular brain development and is known to influence cellular stress responses. To understand the role of Psen1 in endothelial stress responses, we investigated the effects of serum withdrawal on wild type (wt) and Psen1−/− embryonic brain endothelial cells. Serum starvation induced apoptosis in Psen1−/− cells but did not affect wt cells. PI3K/AKT signaling was reduced in serum-starved Psen1−/− cells, and this was associated with elevated levels of phospho-p38 consistent with decreased pro-survival AKT signaling in the absence of Psen1. Fibroblast growth factor (FGF1 and FGF2), but not vascular endothelial growth factor (VEGF) rescued Psen1−/− cells from serum starvation induced apoptosis. Inhibition of FGF signaling induced apoptosis in wt cells under serum withdrawal, while blocking γ-secretase activity had no effect. In the absence of serum, FGF2 immunoreactivity was distributed diffusely in cytoplasmic and nuclear vesicles of wt and Psen1−/− cells, as levels of FGF2 in nuclear and cytosolic fractions were not significantly different. Thus, sensitivity of Psen1−/− cells to serum starvation is not due to lack of FGF synthesis but likely to effects of Psen1 on FGF release onto the cell surface and impaired activation of the PI3K/AKT survival pathway.

Presenilin 1 (Psen1) is a highly conserved multifunctional transmembrane protein involved in early-onset familial Alzheimer’s disease (FAD)^1^. It is an integral component of the γ-secretase complex, which cleaves type 1 single-pass transmembrane proteins within their transmembrane domains, leading to the release of peptides that can have nuclear or non-nuclear signaling functions^1^,^2^. Psen1 also has non-γ-secretase–dependent activity via interactions with other proteins that do not involve proteolytic activity^3^ the best characterized being Psen1’s interaction with β-catenin, an essential component of the Wnt signaling pathway^2^,^4^,^5^,^6^.

Psen1 is crucial for brain development. Psen1-null (Psen1−/−) mutant mice display defects in cortical lamination^7^,^8^. Psen1 also plays roles in vascular development and homeostasis in brain. In Psen1−/− mice, central nervous system (CNS) hemorrhages are observed at mid-gestation^7^,^9^,^10^ in the setting of an aberrant microvasculature characterized by decreased density, less branching, and increased vessel diameter^11^. Transgenic expression of Psen1 using a bacterial artificial chromosome carrying the M146V FAD mutation can rescue the embryonic lethality and neurovascular abnormalities of Psen1−/− mice but an age-dependent vascular degeneration develops in brain that is characterized by a reduced microvasculature, thickening of the vascular basement membranes, and presence of abnormally looped and string vessels^12^.

Using an *in vitro* culture system of differentiating embryonic stem cells, it was shown that Psen1 is involved in the regulation of the growth and differentiation of endothelial progenitor cells through its β-catenin-binding region^13^. Psen1 also regulates levels of extracellular matrix components in the vascular basal membrane^14^. In embryonic brain, Psen1 deficiency in endothelial cells results in decreased turnover of the extracellular matrix protein fibronectin^14^.

Presenilins and presenilin FAD mutants have long been known to influence stress responses in cells including sensitivity to apoptosis^15^,^16^,^17^,^18^,^19^,^20^. To understand the role of Psen1 in endothelial cells, we analyzed the response of embryonic brain endothelial cells to a stress signal generated by serum withdrawal. Serum removal can be used to model apoptosis in endothelial cells^21^,^22^,^23^,^24^,^25^ and causes apoptosis in endothelial cells from various sources including human umbilical vein^26^,^27^,^28^, human foreskin microvasculature^29^, and bovine aorta^30^. In the present study, we show that serum starvation of Psen1−/− brain endothelial cells leads to their detachment from a collagen type IV substrate and apoptosis, but does not significantly affect the viability or attachment of wild-type (wt) brain endothelial cells. Using serum- and supplement-free media we show that either acidic or basic fibroblast growth factors (FGFs) are able to rescue brain endothelial cells from apoptotic cell death following serum starvation, whereas vascular endothelial cell growth factor (VEGF) cannot.

## Results

### Serum starvation induces apoptosis in brain endothelial cells lacking Psen1

**Figure 1:**
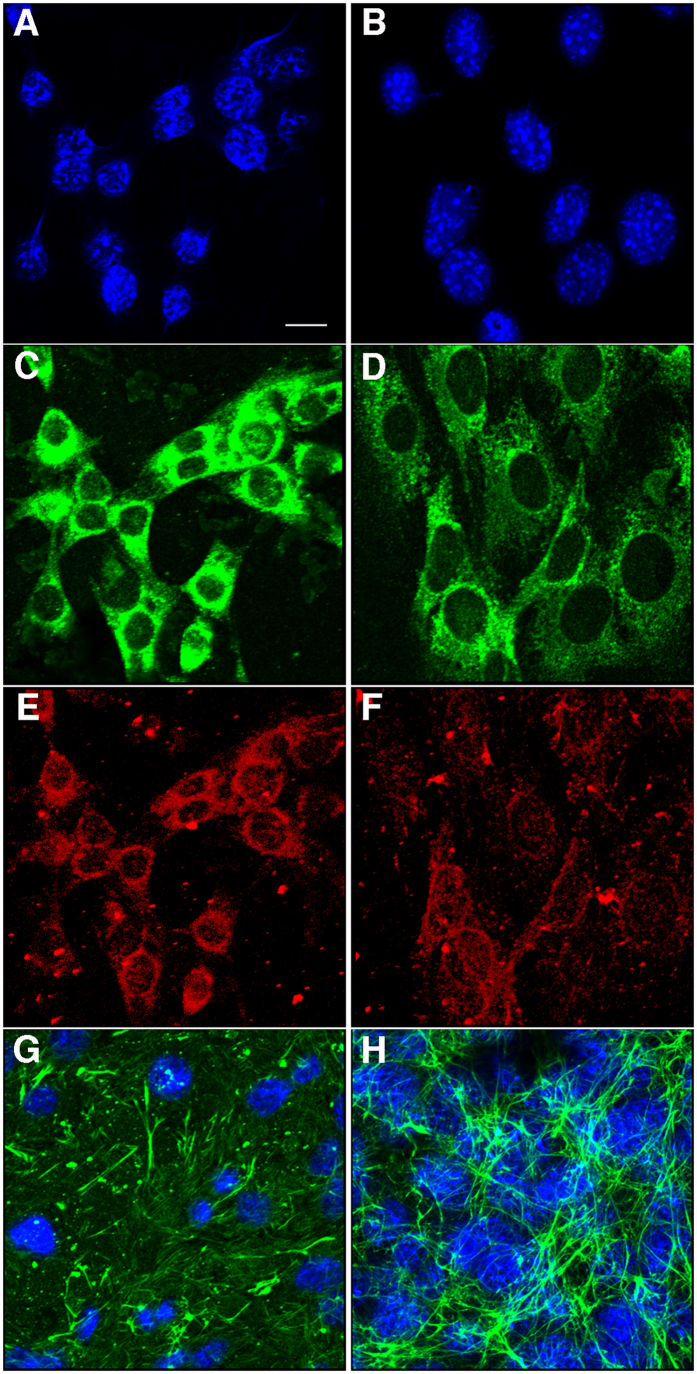
Immunocytochemical characterization of brain endothelial cells. Wt (**A**,**C**,**E**) and Psen1−/− (**B**,**D**,**F**) brain endothelial cells were fixed with acetone/methanol and immunostained for laminin (**C**,**D**) and PECAM (CD31; **E**,**F**) along with a DAPI nuclear stain (**A**,**B**). Panels (**G**,**H**) show confocal images of Wt (**G**) and Psen1−/− (**H**) endothelial cells immunostained for fibronectin (green) with DAPI counterstaining (blue). Scale bar, 10 μm.

Using methodology previously described, endothelial cells were isolated from brains of embryonic day (E)14.5–15.5 wt and Psen1−/− embryos^31^. The wt and Psen1−/− endothelial cells used in this study expressed the endothelial extracellular matrix markers laminin (Fig. 1C,D), platelet/endothelial cell adhesion molecule 1 (PECAM-1; Fig. 1E,F), and fibronectin (Fig. 1G,H). As previously reported^14^, fibronectin was increased in the extracellular matrix of Psen1−/− cells (Fig. 1H).

**Figure 2:**
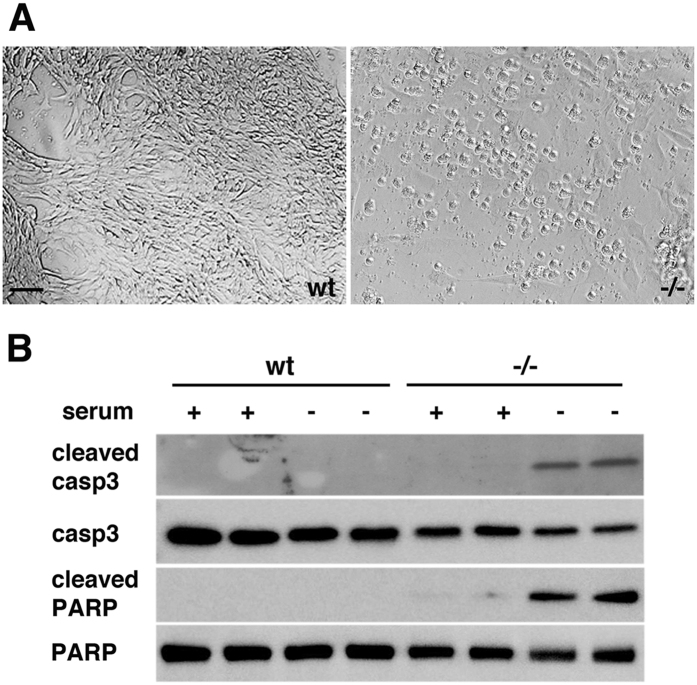
Serum starvation results in apoptosis of Psen1−/− endothelial cells. (**A**) Brightfield micrographs of wt (left) and Psen1−/− cells (right) after serum starvation for 16 h. Note the rounded appearance of the Psen1−/− cells in contrast to the normal appearance of wt cells. Scale bar, 20 μm. (**B**) Western blot detection of cleaved and total caspase 3 (casp3) or PARP from cells grown for 16 h under serum containing (+) or serum starved (−) conditions. Note the presence of cleaved fragments of caspase 3 and PARP, both markers of apoptosis, in serum-starved Psen1−/− cells but not in Psen1−/− cells grown in serum or in wt cells maintained under either condition. The blots are representative of three independent experiments.

Serum deprivation can trigger apoptosis in endothelial cells^26^,^32^. We tested wt and Psen1−/− brain endothelial cells for their ability to withstand serum deprivation. We found that whereas wt brain endothelial cells could withstand serum starvation, Psen1−/− endothelial cells rapidly underwent apoptosis following serum withdrawal. After 12–18 h of serum deprivation, almost all Psen1−/− cells showed apoptotic features becoming generally rounded and frequently detached from the surface of the culture dish, unlike wt cells which maintained their normal appearance (Fig. 2A).

**Figure 3:**
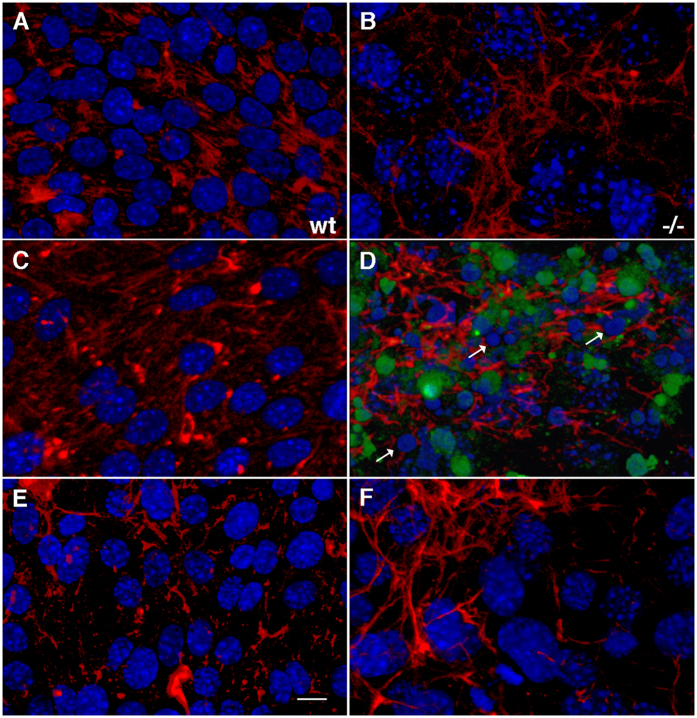
Presence of fragmented nuclei and TUNEL-positive cells among Psen1−/− endothelial cells after serum starvation and rescue by FGF treatment. Wild-type (**A**,**C**,**E**) and Psen1−/− (**B**,**D**,**F**) endothelial cells were grown overnight in ECGM (**A**,**B**), serum-free medium (**C**,**D**), or serum-free medium supplemented with FGF2 (**E**,**F**). The cells were then fixed with 4% paraformaldehyde (PFA) in PBS and immunostained for fibronectin (red) and TUNEL stained (green). Note the presence of TUNEL-stained cells and nuclear fragmentation (arrows) in Psen1−/− cells grown in the absence of serum (**D**). Psen1−/− cells treated with FGF2 (**F**) were normal in appearance and no apoptosis was detectable (compare **F** with **D**). Wt endothelial cells (**A**,**C**,**E**) were not sensitive to serum starvation, and no TUNEL staining or nuclear fragmentation was detectable (**C**). Scale bar, 10 μm.

Western blot analysis of endothelial cells after 16 h of serum starvation showed the presence of activated (cleaved) caspase 3 as well as the cleaved form of its substrate poly(ADP-ribose) polymerase (PARP) in Psen1−/− cells, but not in wt cells (Fig. 2B). Tunel staining confirmed the presence of Psen1−/− apoptotic cells (Fig. 3D). Nuclei in serum-starved Psen1−/− endothelial cells were frequently fragmented, and nuclear chromatin was often condensed (Fig. 3D). In contrast, wild-type cells showed none of these features (Fig. 3C).

### Reduced PI3K/AKT signaling in serum-starved Psen1−/− endothelial cells

**Figure 4:**
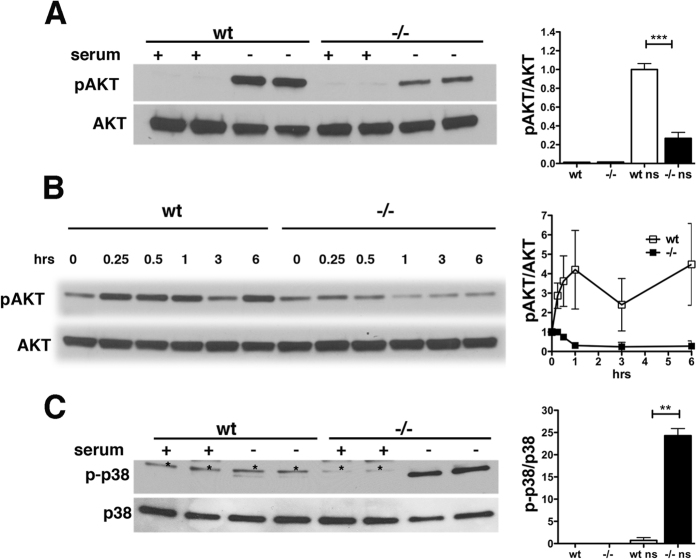
Reduced AKT activation and increased p38 phosphorylation in Psen1−/− cells upon serum deprivation. Psen1−/− and wt cells were grown in the presence (+) or absence (−) of serum, lysed, and analyzed by Western blotting. (**A**) Cells were serum starved for 16 h and AKT phosphorylation was determined with antibodies to phospho-AKT (Ser473) (**A**, upper panel) and total AKT (**A**, lower panel). (**B**) Cells were serum starved for the indicated times (15 min to 6 h) and AKT phosphorylation was analyzed as above. (**C**) Cells were serum starved as in (**A**), and p38 phosphorylation was analyzed with antibodies against phosphorylated p38 (**C**, upper panel) and total p38 (**C**), lower panel). Panels on the right in (**A**,**C**) show quantification of the relative levels of pAKT/total AKT and phospho-p38/total p38. Asterisks in panel in (**B**) indicate a non-specific band. Shown are representative blots from three independent experiments. The panel on the right in (**B**) shows quantification of pAKT levels after different durations of serum deprivation from an experiment that was run in duplicate. Error bars represent standard deviations of the mean. ***p < 0.001, **p < 0.05 indicate statistically different comparisons (unpaired *t*-tests).

The PI3K/AKT pathway is recognized as a key regulator of cell survival and protection against apoptosis in many cell types^33^ including endothelial cells^21^,^34^,^35^,^36^. We examined AKT phosphorylation in endothelial cells after 16 h of serum deprivation. Psen1−/− cells showed significantly less AKT phosphorylation than did wt cells (Fig. 4A). Analysis of the time course of AKT phosphorylation showed that in Psen1−/− cells, pAKT levels rapidly decreased in the first hour and remained low thereafter, whereas pAKT levels in wt cells increased and then remained high (Fig. 4B). p38 MAP kinase also modulates apoptotic cell death in many cell types, and it has been reported that impaired PI3K/AKT signaling leads to activation of p38 and apoptosis^36^,^37^. Because AKT activation was reduced in Psen1−/− cells, we examined p38 phosphorylation in serum-starved cells. Whereas p38 phosphorylation was barely detectable in wt cells after 16 h of serum starvation, Psen1−/− cells showed elevated levels of phosphorylated p38 consistent with decreased pro-survival AKT signaling and an apoptotic phenotype (Fig. 4C).

### FGF but not VEGF prevents apoptotic cell death of Psen1−/− endothelial cells following serum starvation

**Figure 5:**
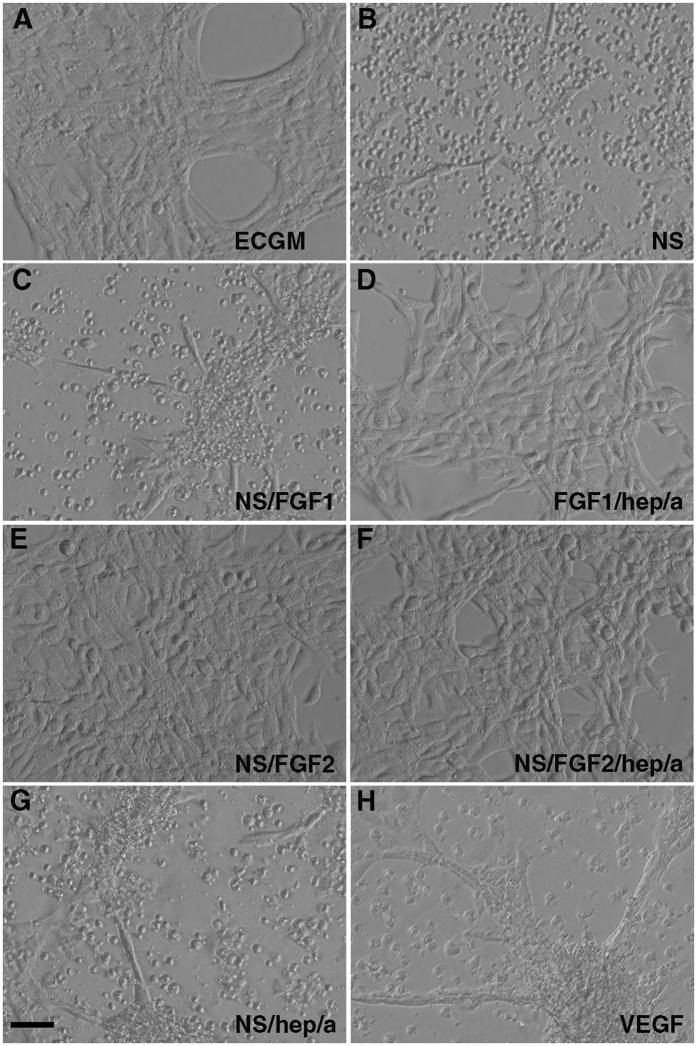
FGF prevents apoptosis of PSEN1−/− endothelial cells grown in the absence of serum. Brightfield micrographs are shown of Psen1−/− cells grown in ECGM (**A**), serum-free media alone (**B**), or serum-free media supplemented with 25 ng/ml FGF1 (**C**), FGF1 with heparin and albumin (**D**), FGF2 (**E**), FGF2 with heparin and albumin (**F**), heparin and albumin (**G**) or VEGF (**H**). Scale bar, 25 μm.

**Figure 6:**
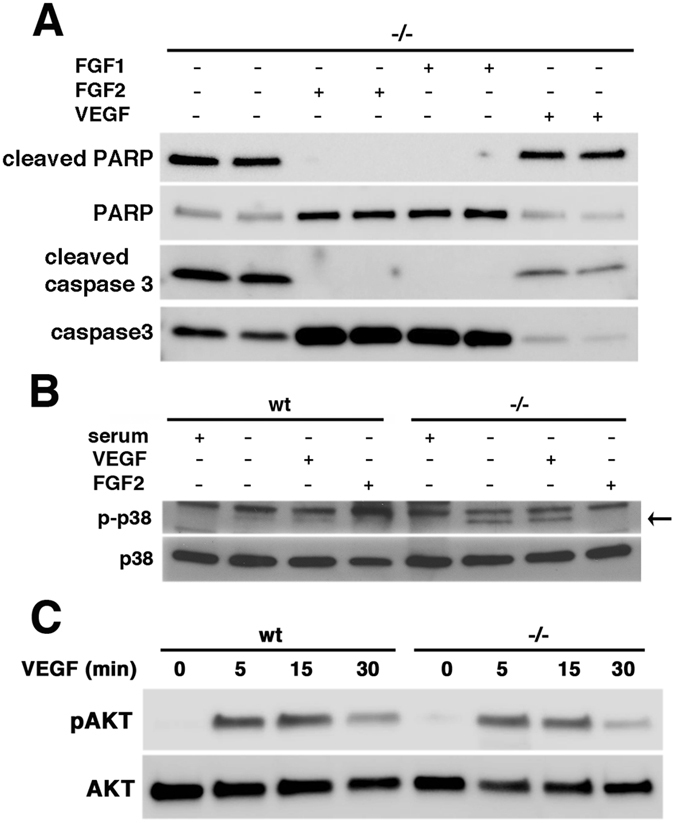
FGF but not VEGF prevents caspase3 and p38 activation in serum-starved Psen1−/− cells. Cells were grown in serum-free medium alone or serum-free medium supplemented with FGF1, FGF2, or VEGF for 16 h, lysed, and analyzed by Western blotting. (**A**) Psen1−/− cells were analyzed for cleaved PARP and total PARP (**A**, top panels), and cleaved caspase 3 and total caspase 3 (**A**, bottom panels). (**B**) p38 phosphorylation was examined in wt and Psen1−/− cells treated with FGF2 or VEGF. The lower band indicated by the arrow corresponds to phosphorylated p38 (p-p38, **B** upper panel). The upper band present in all lanes is non-specific. (**C**) Cells were treated with VEGF (25 ng/ml) for the indicated times following 3 h of serum starvation and phosphorylation of AKT was analyzed in cell lysates using antibodies against phospho-AKT (Ser473) and total AKT. In each panel representative blots are shown from three (**A**,**B**) or two independent experiments (**C**).

FGF2 has been reported to protect endothelial cells from the apoptotic effects of serum starvation^22^,^26^. We investigated whether FGF could protect Psen1−/− cells from apoptosis induced by serum deprivation. As shown in Fig. 5, basic FGF2 as well as acidic FGF1 at concentrations of 25 ng/ml in serum-free media prevented apoptosis induced by serum-deprivation in Psen1−/− endothelial cells (compare Fig. 5D–F to cells grown without serum in 5B and cells grown in complete endothelial cell growth medium [ECGM] in 5A). FGF1 was inactive when added in the absence of albumin and heparin (Fig. 5C), whereas FGF2 was active in both conditions (Fig. 5C,D). Moreover, Psen1−/− endothelial cells treated with FGF2 in the absence of serum no longer exhibited TUNEL staining and showed no nuclear fragmentation as visualized by DAPI staining (Fig. 3F, compare to cells grown in the absence of serum in Fig. 3D or cells grown in ECGM in Fig. 3B). These results were confirmed by Western blot analysis, in which caspase 3 (Fig. 6A, upper panels), cleaved PARP (Fig. 6A, lower panels), and p38 activation (Fig. 6B) were not detectable in Psen1−/− endothelial cells.

In human umbilical vein endothelial cells (HUVEC), VEGF has been reported to induce a survival response in serum-free medium and to be a primary survival factor via activation of the PI3K/AKT pathway^21^. To determine the effects of VEGF on Psen1−/− cells, AKT phosphorylation was measured following VEGF treatment. As shown in Fig. 6C, the response of Psen1−/− endothelial cells to VEGF was similar to that of wt cells. However, VEGF treatment (25 ng/ml) failed to prevent apoptosis of Psen1−/− brain endothelial cells under serum starvation conditions as indicated by the typical apoptotic phenotype of cells (Fig. 5H) and the presence of cleaved caspase 3, cleaved PARP, and activated p38 (Fig. 6A,B).

### Blocking FGF signaling induces apoptosis in wt endothelial cells following serum starvation

**Figure 7:**
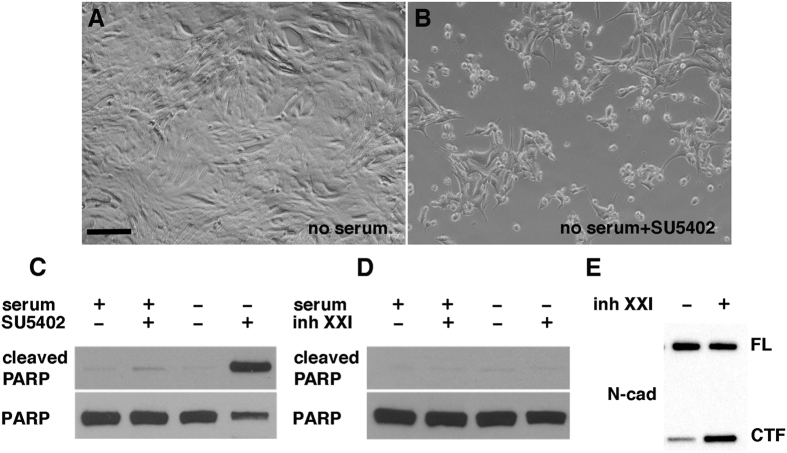
Inhibition of FGF signaling renders wild-type cells sensitive to apoptosis by serum deprivation. Wild-type endothelial cells were treated with SU5402 (an inhibitor of FGF signaling) and then serum starved for 16 h. Brightfield images are shown of cells grown without pretreatment with 30 μM SU5402 (**A**) or with pretreatment (**B**) before serum starvation. (**C**) Western blot analysis of cleaved PARP in wild-type endothelial cells pretreated (+) or not (−) with SU5402 and (**D**) absence of cleaved PARP in wild-type endothelial cells pretreated for 16 h with 10 μM inhibitor XXI and then serum starved for an additional 16 h. (**E**) Western blotting shows accumulation of the N-cadherin C-terminal fragment (CTF) following treatment with γ-secretase inhibitor XXI. Scale bar in A, 25 μm.

To confirm the essential role of FGF in preventing apoptosis in response to serum deprivation, Psen1 wt cells, which are normally resistant to serum starvation, were treated overnight with 30 μM SU5402, an irreversible antagonist of the FGFR-specific tyrosine kinase^38^, and serum-starved for 16 h. Pretreatment of wt cells with inhibitor followed by overnight serum starvation resulted in apoptosis as indicated by cell morphology (Fig. 7A,B) and the presence of cleaved PARP (Fig. 7C). Although SU5402 can also inhibit VEGFR^39^, it is likely that apoptosis was caused by inhibition of FGF signaling given that VEGF failed to prevent serum deprivation-induced apoptosis in Psen1−/− endothelial cells.

### Blocking γ-secretase activity does not sensitize wt endothelial cells to apoptosis following serum starvation

One of the main biological functions of Psen1 is its role as a component of the γ-secretase complex^1^,^2^. Therefore, we investigated whether inhibition of γ-secretase activity could affect survival of wt endothelial cells in the absence of serum. As shown in Fig. 7D, pretreatment of wt endothelial cells for 16 h with the γ-secretase inhibitor XXI followed by serum starvation did not result in apoptosis as judged by cleavage of PARP. Accumulation of N-cadherin C-terminal fragment (Fig. 7E) confirmed that the inhibitor blocked γ-secretase activity.

### Localization and levels of FGF2 in endothelial cells

**Figure 8:**
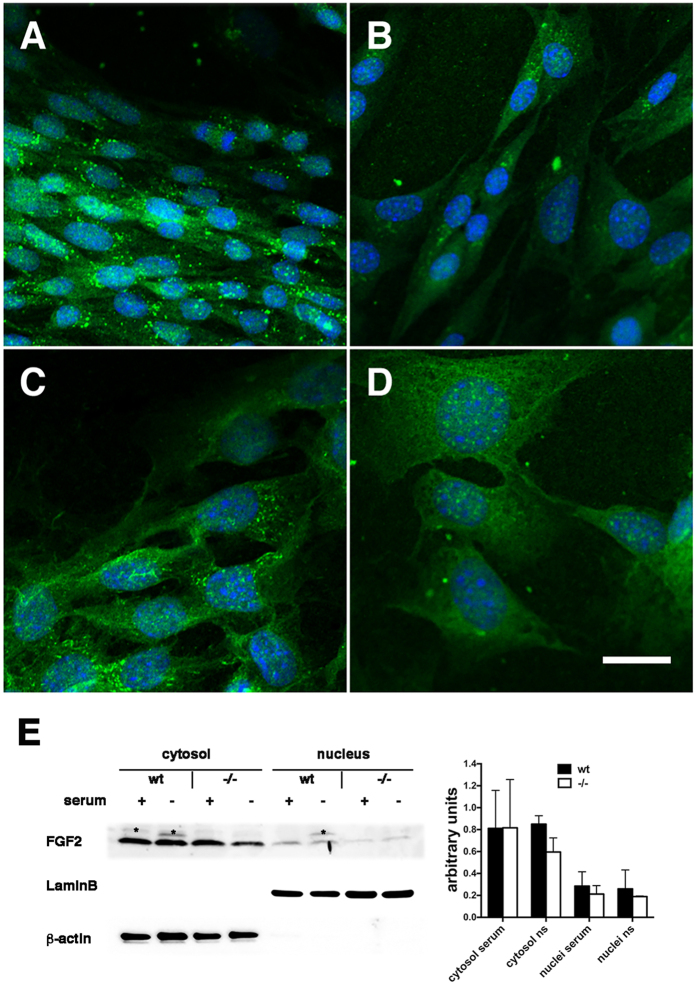
Intracellular localization and levels of FGF2 in the presence and absence of serum. Wt and Psen1−/− cells were grown in the presence (ECGM) or absence of serum for 6 h, fixed in 4% paraformaldehyde in PBS, and immunostained with a rabbit anti-FGF2 polyclonal antibody (**A**–**D**). Shown are confocal images of wt endothelial cells grown with serum (**A**) or without serum (**B**) and Psen1−/− endothelial cells grown with serum (**C**), or without serum (**D**). Scale bar 20 μm. Western blot analysis of FGF2 in cytosolic and nuclear fractions from wt and Psen1−/− endothelial cells grown in the presence or absence of serum (**E**). Lamin B and β-actin were used as control markers for the nuclear and cytosolic fractions, respectively. Asterisks indicate non-specific bands. The blot is representative of two independent experiments. The graph on the right shows quantitative data for the two experiments.

FGF2 is present in multiple subcellular locations depending on the cell type and state of cells, which influences its biological effects^40^. Therefore we investigated FGF2 distribution in wt and Psen1−/− cells in the presence of serum and after 6 h of serum deprivation via immunocytochemical staining. In the presence of serum, FGF2 immunoreactivity in both wt and Psen1−/− cells was largely concentrated in nuclear and cytoplasmic vesicles (Fig. 8A,C). In the absence of serum, FGF2 staining became more diffuse especially in the cytosol, although some vesicles could still be observed (Fig. 8B,D) without any differences between wt and Psen1−/− cells.

As an additional indication of the levels and intracellular distribution of FGF2 cytosolic and nuclear fractions were prepared from control and serum-starved wt and Psen1−/− endothelial cells and FGF2 was captured with heparin–agarose beads. Western blot analysis of the FGF2-enriched fractions did not show any significant differences in the levels of FGF2 in the cytosolic and nuclear fractions of wt and Psen1−/− cells, whether in the presence or absence of serum, when FGF2 levels were normalized to those of β-actin (cytosolic) or lamin B (nuclear) (Fig. 8E).

## Discussion

Inhibition of apoptosis and endothelial cell survival is essential during angiogenesis and for maintenance of blood vessel integrity, and is favored by many growth factors as well as integrin-mediated adhesion to the extracellular matrix^34^. Apoptosis of endothelial cells, however, occurs as part of vessel regression and remodeling and is most likely triggered by loss of growth factors^41^,^42^. *In vitro* serum deprivation triggers apoptosis in endothelial cells from different sources, but serum deprivation-induced apoptosis can be suppressed by extracellular survival signals promoted by growth factors such as VEGF or FGF^41^.

Our results with brain endothelial cells isolated from wt and Psen1−/− embryonic brains show that Psen1-deficient cells, when deprived of serum, commit to apoptosis, whereas wt cells are resistant to the effects of serum withdrawal. In contrast to wt cells, activation and maintenance of PI3K/AKT-mediated signaling in Psen1−/− cells was impaired and insufficient to withstand serum deprivation. This pathway is considered a major pro-survival pathway for cells as it provides signals to respond to and overcome apoptotic stimuli such as serum deprivation^33^. The possible involvement of Psen1 in PI3K/AKT signaling has been suggested previously in studies which found that cultured fibroblasts and neurons show reduced levels of basal pAKT under normal growth conditions^43^,^44^. Moreover, Psen1−/− neurons show a progressive reduction in basal pAKT as they mature, which is associated with activation of caspase 3 and apoptosis^44^. In endothelial cells, the presence of Psen1 appears to have protective effects under stress conditions such as serum deprivation. This effect was not associated with Psen1 γ-secretase activity, as treatment of wt cells with a γ-secretase inhibitor did not result in apoptosis. This is consistent with the results of other studies in fibroblasts and neurons showing that Psen1’s effect on AKT signaling is γ-secretase-independent^43^,^44^.

Previous reports found that apoptosis induced by serum deprivation of endothelial cells can be prevented by FGF, VEGF, or phorbol-12, myristate-13 acetate^21^,^22^,^26^,^32^,^35^. In the case of the Psen1−/− endothelial cells, we found that both FGF1 and FGF2 prevent apoptosis in serum-free conditions. VEGF, however, unlike in other endothelial cells types^21^,^35^, could not rescue Psen1−/− cells from apoptosis. Studies in HUVECs and bovine aortic endothelial cells have shown that VEGF can have proapoptotic effects when the PI3K/AKT signal is blocked^36^. Because Psen1−/− endothelial cells have impaired PI3K/AKT activation when subjected to serum starvation, it might be that under these conditions VEGF in fact favors apoptosis.

How FGF2 prevents apoptosis is unclear. FGF2 activates PI3K/AKT signaling making one possible mechanism that exogenously added FGF2 stimulates PI3K/AKT signaling sufficiently that heightened growth factor stimulation can overcome deficient PI3/AKT activation in Psen1−/− cells. However, if direct effects on PI3K/AKT signaling explain the prosurvival actions of FGF2 it is curious that VEGF, which also activates PI3K/AKT signaling^21^, did not rescue. FGF2 activates other signaling pathways including MAPK. In HUVECs, FGF2 also appears to act independently of its mitogenic effects and activation of MAPK to promote cell attachment^22^.

Another study in HUVECs showed the existence of an FGF2 autocrine loop by which cells can become more resistant to serum deprivation by increasing the amount of FGF2 on the cell surface^45^. Under normal conditions, FGF is supplied to endothelial cells in culture by serum and endothelial cell growth supplement, a poorly defined extract from bovine neural tissues known to contain FGF1 and FGF2^46^,^47^. Therefore, it is possible that under conditions of serum starvation Psen1−/− cells cannot compensate in an autocrine fashion for the loss of the exogenous FGF.

FGF2 is synthesized primarily as its 18-kDa low molecular weight form, and, via alternative initiation codons, higher molecular forms are made that are only intracellular and remain in the cytosol and nucleus^40^. The 18-kDa FGF2 is transported outside the cell by an unconventional secretion pathway that does not involve the endoplasmic reticulum/Golgi-mediated secretion pathway but rather direct translocation across the plasma membrane in a process involving phosphatidylinositol biphosphate (PIP2), ATP1A1, and Tec 1 kinase^48^,^49^. Translocated FGF2 is not secreted into the medium but retained at the cell surface by binding to heparan sulfate proteoglycan which acts as an extracellular trap and results in FGF2 acting in a paracrine or local manner near the source of its expression^48^,^49^.

FGF2 signaling is activated by its interaction with FGFR on the cell surface. However, FGF2 can also be taken up by receptor-mediated endocytosis into the cytosol and transported from intracellular vesicles to the nucleus. This translocation appears to be important for eliciting the mitogenic activity of FGF2^40^. Previous studies in HUVECs^45^ showed that the 18-kDa form was responsible for protection from apoptosis under serum withdrawal conditions. Therefore, absence of Psen1 could impair the transport of FGF across the membrane. In fact, we have observed that although after 16 h of serum starvation most Psen1−/− cells undergo apoptosis, a few cells can survive. If maintained in the apoptotic conditioned media the surviving cells are eventually able to recover. This supports the idea that in Psen1−/− cells under serum starvation the release of FGF may occur at a much reduced rate, taking longer to build up the FGF2 signaling necessary to activate the PI3K/AKT pathway to promote survival. As for wild type cells, their resistance to serum deprivation is likely due to the fact that they can rapidly compensate for the loss of exogenous FGF by releasing sufficient endogenous FGF to keep the survival pathway active and prevent apoptosis from occurring.

## Conclusions

Serum starvation is routinely used as a tool to study molecular mechanisms involved in protein degradation, cellular stress responses, autophagy, and apoptosis under well-defined normal and pathological conditions. In the present study, we found that serum starvation of brain endothelial cells deficient for Psen1 induces cellular apoptosis and is associated with impairment of pro-survival PI3K/AKT signaling. In contrast, cells expressing Psen1 were insensitive to serum starvation and displayed normal PI3K/AKT signaling. The protective effect of Psen1 expression upon serum starvation was independent of its γ-secretase function. We also showed that addition of FGF1 or FGF2 in the serum-free medium entirely prevents apoptosis in Psen1−/− brain endothelial cells. Analysis of FGF2 by immunostaining and Western blot showed that FGF2 levels and distribution in endothelial cells did not differ significantly in the presence or absence of serum in either cell type. This suggests that the sensitivity of Psen1−/− endothelial cells to serum deprivation is not due to changes in the intracellular FGF2 levels or localization within the cell.

FGF2 may rescue Psen1−/− endothelial cells from apoptosis through its ability to activate PI3K/AKT signaling. FGF2 is also an unusual protein that is not secreted directly into the medium but released through a non-canonical mechanism that involves translocation through the cell membrane and retention at the cell surface mainly by heparan sulfate proteoglycans. If Psen1 plays a role in this translocation process in the absence of serum Psen1−/− cells may fail to release sufficient endogenous FGF to the cell surface, a defect that can be compensated for by the provision of exogenous FGF.

## Methods

### Coating of culture surfaces

Polystyrene Nucleon plasticware (6- and 12-well dishes) and Permanox chamber slides (Nunc, Roskilde, Denmark) were coated with a solution of 10 μg/ml mouse collagen type IV (BD Biosciences, Bedford, MA) in 50 mM HCl (0.14 ml/cm^2^) and air dried for 48–72 h in a sterile biological safety cabinet. Collagen type IV-coated plasticware was stored in sealed bags at −20 °C until use.

### Preparation of embryonic cerebral vascular endothelial cells

Endothelial cells were isolated from E13.5–14.5 embryonic telencephalic and mesencephalic brain regions and grown on collagen type IV-coated plasticware in ECGM, which consisted of Dulbecco’s Modified Eagle Medium/Ham’s F12 (DMEM-F12) supplemented with 10% heat-inactivated horse serum, 10% heat-inactivated fetal calf serum, 100 μg/ml endothelial cell growth supplement, and 100 μg/ml heparin^31^. Cells were always kept at high densities and passaged at a 1:2 dilution. The endothelial phenotype of mouse embryonic endothelial cells was verified by immunocytochemical staining with antibodies against PECAM-1, fibronectin, and laminin as previously described previously^31^.

### Serum starvation, growth factors, and inhibitors

Cultured endothelial cells were rinsed 3 times with Hanks balanced salt solution (HBSS) and serum-starved in either DMEM alone or DMEM containing 0.1% bovine serum albumin (BSA, fraction V) and 100 μg/ml heparin. FGF1, FGF2, and VEGF (R&D Systems, Minneapolis, MN) were added individually at 25 ng/ml in DMEM-BSA-heparin media along with the γ-secretase inhibitor XXI (compound E, 10 μm, EMD Millipore, Billerica, MA) or the FGFR antagonist SU5402 (10 μm, Santa Cruz Biotechnology, Santa Cruz, CA). In some experiments, FGF1 and FGF-2 were added in DMEM alone without BSA and heparin.

### Western blotting

Endothelial cells (attached and unattached cells in the case of apoptotic cells) were lysed in 10 mM sodium phosphate, pH 7.4, 150 mM NaCl, 1 mM EDTA, 1% Triton, 0.5% Na deoxycholate, and 0.5% sodium dodecyl sulfate (SDS) supplemented with a protease and phosphatase inhibitor cocktail (Pierce, Rockford, IL). Protein concentration was determined with the BCA reagent (Pierce). Aliquots of the lysates (10 μg protein) were separated by SDS-polyacrylamide gel electrophoresis (PAGE) and blotted onto polyvinyl difluoride (PVDF) membranes. Membranes were blocked for 1 h in Tris buffer saline/1% Tween-20 (TBST) containing 0.5% non-fat dry milk and incubated overnight at 4 °C with the appropriate primary antibody diluted in the above blocking solution or in TBST/5% BSA. After washing with phosphate-buffered saline (PBS), the membrane was incubated with anti-rabbit IgG horseradish peroxidase-conjugated secondary antibodies (GE Healthcare, Piscataway, NJ) and the bands visualized with the ECL Prime reagent (GE Healthcare). The membranes were exposed to film or imaged with the Amersham Imager 600 (GE Healthcare). Quantitation was performed with Image Quant software (GE Healthcare). Statistical analysis was performed as previously described^14^. Primary antibodies against the following targets were used: cleaved caspase 3 (1:1000), caspase 3 (1:1500), cleaved PARP (1:1500), phospho-p38 (T180/Y182) (1:1000), total p38 (1:1000), phospho AKT (S473) (1:1200), total AKT (1:2000) (all from Cell Signaling, Danvers, MA), and total PARP (1:1500) obtained from Abcam (Cambridge, MA).

For FGF2 analysis EC cells were serum-starved for 6 h and harvested along with control cells that were not serum-starved by scraping with a rubber policeman. Cytosol and nuclear fractions were isolated using the N-Per reagent (Pierce) as described by the manufacturer’s instruction. Protein concentrations were determined by the BCA methods as above. FGF2 was captured with heparin-agarose beads^45^. Aliquots of the cytosolic and nuclear fractions (200–250 μg total protein) were diluted in 50 mM Tris HCl pH 7.4, 0.5 M NaCl and incubated overnight at 4 °C with 30 μl of heparin-agarose beads (Sigma-Aldrich, St Louis, MO) equilibrated with the same buffer. The beads were then washed three times with the above buffer and the captured FGF2 eluted by boiling in SDS-PAGE sample loading buffer. The samples were separated by SDS PAGE, blotted, and analyzed as above using rabbit polyclonal anti-FGF2 antibodies (1:2000) (Abcam, Cambridge, MA). The cytosol and nuclear fractions were analyzed by Western blot using a rabbit polyclonal anti-lamin B (1:1000, Proteintech, Rosemont IL) and a mouse monoclonal against beta-actin (1:2000, Sigma Aldrich, St Louis, MO) as nuclear and cytosolic markers, respectively.

### Immunocytochemistry and TUNEL assay

Endothelial cells grown on collagen IV-coated slides were fixed with acetone/methanol or 4% paraformaldehyde in phosphate-buffered saline (PBS). The slides were blocked for 1 h in TBS containing 0.3% Triton X-100 and 10% normal goat serum and incubated overnight at room temperature with primary antibodies diluted in the above blocking solution. Slides were washed with PBS and incubated for 2 h with the appropriate secondary antibodies conjugated to Alexa Fluor 488 or 562 (Molecular Probes-ThermoFisher, Grand Island, NY) before being washed with PBS and mounted with Fluorogel mounting medium (EMS, Hatfield, PA,). The following antibodies were used: rabbit polyclonal anti-laminin (1:100) and rabbit polyclonal anti-fibronectin (1:400; both from Sigma-Aldrich), rat monoclonal anti-PECAM (CD-31, 1:100; BD Biosciences, San Jose, CA), and rabbit polyclonal anti-FGF2 (1:300; Abcam). Terminal deoxynucleotidyl transferase dUTP (TUNEL) staining was performed using the *In Situ* Cell Death Detection Kit (Roche Life Science, Indianapolis, IN) according to the manufacturer’s instructions.

## Additional Information

**How to cite this article**: Gama Sosa, M. A. *et al*. Fibroblast growth factor rescues brain endothelial cells lacking presenilin 1 from apoptotic cell death following serum starvation. *Sci. Rep.* **6**, 30267; doi: 10.1038/srep30267 (2016).
